# Environmental drivers of Arctic communities based on metabarcoding of marine sediment eDNA

**DOI:** 10.1098/rspb.2023.1614

**Published:** 2024-01-24

**Authors:** Nathan R. Geraldi, Dorte Krause-Jensen, Sarah B. Ørberg, Larissa Frühe, Mikael K. Sejr, Jørgen L. S. Hansen, Lars Lund-Hansen, Carlos M. Duarte

**Affiliations:** ^1^ Red Sea Research Center (RSRC) and Computational Biosciences Research Center (CBRC), King Abdullah University of Science and Technology (KAUST), Thuwal, Saudi Arabia; ^2^ Department of Ecoscience, Aarhus University, Aarhus, Denmark; ^3^ Arctic Research Centre, Aarhus University, Aarhus, Denmark; ^4^ Department of Biology, Aarhus University, Aarhus, Denmark; ^5^ Department of Ecoscience, Aarhus University, Roskilde, Denmark

**Keywords:** Arctic, community ecology, metabarcoding, environmental DNA, indicator species, 18S rRNA

## Abstract

Our ability to assess biodiversity at relevant spatial and temporal scales for informing management is of increasing importance given this is foundational to identify and mitigate the impacts of global change. Collecting baseline information and tracking ecological changes are particularly important for areas experiencing rapid changes and representing data gaps such as Arctic marine ecosystems. Environmental DNA has the potential to provide such data. We extracted environmental DNA from 90 surface sediment samples to assess eukaryote diversity around Greenland and Svalbard using two separate primer pairs amplifying different sections of the 18S rRNA gene. We detected 27 different phyla and 99 different orders and found that temperature and the change in temperature explained the most variation in the community in a single linear model, while latitude, sea ice cover and change in temperature explained the most variation in the community when assessed by individual non-linear models. We identified potential indicator taxa for Arctic climate change, including a terebellid annelid worm. In conclusion, our study demonstrates that environmental DNA offers a feasible method to assess biodiversity and identifies warming as a key driver of differences in biodiversity across these remote ecosystems.

## 1. Introduction

The Arctic Ocean is one of the fastest-warming regions on Earth with three times higher warming rates compared with the global mean [1–3] and can act as a warning example for other ecosystems with lower rates of warming but with long-term warming projections [2]. Warming in Arctic coastal ecosystems leads to declining sea ice coverage especially during the summer and elevated freshwater input from glacier melting [4–6]. These changes can directly alter many ecological processes such as primary production and carbon cycling, cause compositional and functional shifts in Arctic marine communities [7,8], and potentially the extirpation of endemic species [9]. Most studies to determine effects of climate change in the Arctic have focused on terrestrial ecosystems or marine mammals [10,11] although other marine biota in the Arctic Ocean are also sensitive to climate change [12,13]. And while our understanding of Arctic marine flora and fauna is improving, we still lack a broad-scale understanding of the distribution of species and communities because of the difficulty in sampling the Arctic Ocean [14].

Use of environmental DNA (eDNA) enables the detection of a broad array of taxa within targeted taxa groups or even whole domains, such as all eukaryotic organisms [15,16], and taxonomic expertise is not required since the taxonomic assignment of sequences is done via alignments with reference databases. In addition, sample collection can be conducted with limited instruction which gives eDNA-based studies the ability to sample large areas with less resources than traditional sampling. Surveys based on eDNA metabarcoding provide insights into biodiversity and community composition by linking taxonomy to high-throughput sequencing of eDNA [17]. Although definitions of eDNA vary, we consider eDNA to be any DNA extracted from environmental samples including extracellular DNA and DNA within living cells [18]. The feasibility of eDNA metabarcoding for assessments of Arctic marine ecosystems has been proven by recent localized studies [19–21].

Surveys that assess communities across large areas can enable a space-for-time analyses. Space-for-time analyses can allow aspects of general biodiversity like species richness, distribution patterns and compositional turnover to be projected assuming specific variables through space overlayed with these same variables through time [22–24]. This analysis can be particularly insightful where consistent sampling and data gathering through time is difficult, such as in marine Arctic environments. Hence, inferences about changes in Arctic communities with warming can potentially be made based on relationships between changes in the community and environmental variables through space in combination with known or predicted changes in the environmental variables. For example, communities associated with colder northern areas will likely be replaced by communities of warmer southern areas depending on the community's association with temperature and the area-specific warming projections. For example, the productivity and depth range of Arctic kelp tend to increase with longer ice-free periods, and the potential distribution area of kelps has increased and is projected to continue to do so in a warming Arctic [25,26].

Large-scale surveys that encompass environmental gradients can also be used to identify potential bioindicator taxa, which is particularly relevant to the quickly warming Arctic. Identifying bioindicator taxa such as species that cannot tolerate rising temperatures can give early warning to the impacts of warming and could help forecast changes if these species have known effects on food webs or communities, as well as broader environmental changes if they are ecosystem engineers [27,28]. Similar to space-for-time analysis, identifying and then monitoring for indicator taxa can be enhanced using eDNA-based surveys given the ease of sample collection.

This study aimed to: (1) determine the feasibility of using sediment eDNA to assess the Arctic flora and fauna on a broad spatial scale around the Arctic and, if successful, (2) use the results as baseline information on Arctic eukaryote biodiversity and (3) identify main drivers of the spatial patterns in biodiversity and potential indicator taxa to help forecast changes. We used two eukaryotic primer pairs (18S V9&V7) to measure diversity from eDNA extracted from 90 sediment samples spanning in latitude from 64° to 79°N. We then combined the diversity data with environmental variables to assess links between community changes and these variables, as well as to identify indicator taxa associated with specific variables. Linking community changes with environmental variables also allowed us to forecast potential future changes based on space-for-time substitution.

## 2. Methods

### (a) Sample collection

**Figure 1.**
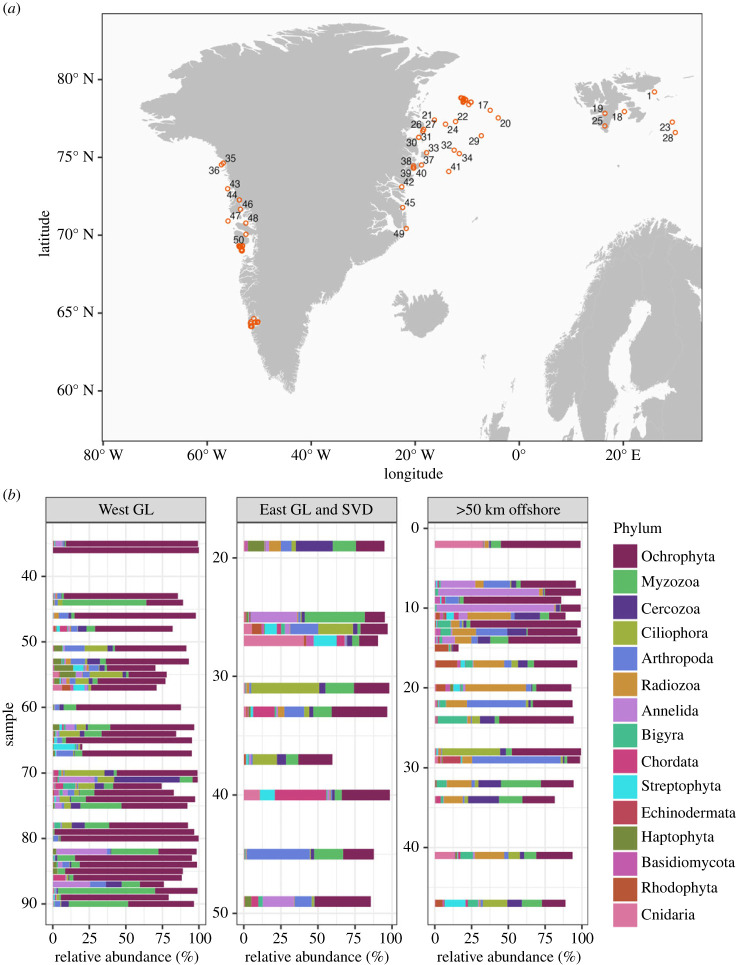
Sample locations around Greenland and Svalbard (*a*), as well as the relative abundance of the most prevalent phyla for locations on the west coast of Greenland, locations on the east coast of Greenland and near Svalbard, or any location more than 50 km from a coastline (*b*). Samples were numbered from north to south as shown in *a* and this labelling is followed in the *y*-axis of *b*. Blank spaces along the *y*-axis were kept to maintain north to south sample labels.

The study included a total of 90 surface sediment samples from marine areas off the west Greenland coast at 64–75°N, the east Greenland coast at 70–79°N and Svalbard at 76–79°N (figure 1*a*). The samples represented water depths from the shore to maximum depths of 1460 m. Samples were collected specifically for this study, as well as taken from existing sediment samples that were collected and preserved following methods appropriate for eDNA studies. Sampling and conservation methods are specified in electronic supplementary material, table S1. Collection methods depended on location characteristics. For example, samples from intertidal sites were collected by a hand-held sampler, shallow sites were collected by self-contained underwater breathing apparatus (SCUBA) divers and deep sites were collected from a research vessel with a box or gravity corer. All collection material that came in contact with samples was sterilized beforehand with 20% bleach and care was taken to collect sediment greater than 2 cm away from material that was not sterilized such as the inner surface of cores. After collection, sediment samples were either frozen or preserved by mixing with an equal volume of either RNAlater or DNAguard.

### (b) DNA extraction

We used the DNA extraction protocol from Lever *et al*. [29], which targets extracellular DNA. This protocol was used because it extracts DNA consistently from different types of sediments [30]. Extraction blanks (nuclease-free water) were included to identify DNA introduced by extraction methods (reagents and consumables) during extraction and one extraction blank was run for every time a different batch of samples was extracted (12 samples).

### (c) DNA amplification and sequencing

This study used two different primer pairs (electronic supplementary material, table S1) that target eukaryotes and amplify sections within the 18S rRNA gene, one of the V7 region [18,31] and the other of the V9 region [32]. The primers contained an Illumina adapter (electronic supplementary material, table S2). A final volume of 10 µl was used for the PCR reactions with 5 µl of Qiagen multiplex PCR master mix (QIAGEN, Valencia, CA, USA), 3.4 µl of PCR grade water, 0.3 µl of 10 mM of primers (forward and reverse), and 1 µl of extracted DNA. PCR settings for the primer pairs are detailed in electronic supplementary material, table S2. We ran five replicate PCR assays for each sample (five different 96-well plates), which were then pooled to reduce potential PCR biases. Two PCR blanks and two positive blanks were run for each primer pair. The positive blanks consisted of relevant DNA extracted from organisms that were targeted by the primers. Two of the eight extraction controls were chosen to sequence with the highest DNA concentration (Qubit 2.0 Fluorometer; Invitrogen, Carlsbad, CA, USA). PCR products were visualized with gel electrophoresis (1.5%) and products were discarded if PCR controls were visible and PCR steps were conducted with all new reagents. Products were then cleaned with AMPure XP magnetic bead-based purification (Beckman Coulter, Brea, CA, USA) following the MiSeq library preparation guide. Primer pairs were sequenced separately on plates containing 90 samples and six control/blanks. In order to maximize the spatial extent, which was a goal of the study, we did not sequence any replicates, such as biological, extraction or PCR replicates.

A second PCR step attached dual indexes and Illumina sequencing adapters from the Illumina Nextera XT index Kit v2 (Illumina, Inc.) to the amplicon using Kapa HotStart HiFi 2x ReadyMix DNA polymerase (Kapa Biosystems Ltd., London, UK). The protocol followed the MiSeq library preparation guide (12.5 µl of polymerase, 5 µl of PCR grade water, 2.5 µl of each index and 5 µl of sample run for 95°C for 3 min, then eight cycles of 95°C for 30 s, then 55°C for 30 s, then 72°C for 30 s and a final stage of 72°C for 5 min). Products of the second PCR were checked for appropriate length (gel electrophoresis) and cleaned using AMPure XP beads. DNA concentrations and size of the amplicons were assessed with a Qubit 2.0 Fluorometer and Tape station system (Agilent Technologies, Santa Clara, USA). The pooled library was created with equimolar amounts of each sample. A maximum of 50 µl of sample was added to the pool to minimize dilution. After pooling, the 18S-V9 amplicon pool was cleaned using Wizard SV Gel and PCR Clean-Up System (Promega, USA) to remove primer dimer and PCR artefacts. DNA concentration of pooled libraries was measured with KAPA SYBR FAST Universal qPCR kit with Illumina Primer Premix (Kapa Biosystems Ltd., London, UK) and the mean DNA length was measured with a Bioanalyzer (Agilent Technologies, Santa Clara, USA). Amplicon libraries were sequenced with 6 picomolar (pM) of product and 20% PhiX control on one lane of an Illumina MiSeq platform at the KAUST core laboratory. Two × 300 bp overlapping paired-end reads were sequenced following the MiSeq library preparation guide and a MiSeq reagent kit v3.

### (d) DNA post-sequence procedures

The post-sequencing workflow and analyses, except demultiplexing and primer removal, were conducted in R version 4.2.2 (R Development Core Team, 2012). Demultiplexing followed the Illumina guide and CUTADAPTv3.0 was used to remove the primers, which allowed one error for every 10 bp in the primer sequence [33]. Next, sequences were improved by correcting substitution errors, filtered and dereplicated following the DADA2 workflow using the *DADA2* package version 1.26.0 [34]. The DADA pipeline was more accurate and resulted in fewer spurious reads than other commonly used pipelines based on clustering similar reads [35]. Unique reads are referred to as Amplicon Sequence Variants (ASV). Primer pairs were run through the pipeline individually. Specifically, the DADA2 pipeline included filtration of forward and reverse reads (filterandtrim function; parameters: truncLen = c(105 105) for V7 and c(150 150) for V9, maxN = 0, maxEE = c(2,2), truncQ = 2, rm.phix = T), modelled errors (*learnerrors* function), paired and de-replicated (derepfastq function).

The *dada* function determined the ASVs using all samples within a MiSeq run. Identical reads were combined (*mergePairs* function), and sequences were removed if beyond the target amplicon size, from 100 to 150 bp for V7 and 150 to 200 bp for V9 primer pairs. Finally, chimera sequences were identified and removed with the *removeBimeraDenovo* function. Unique ASVs were assigned taxonomy with the ribosomal database project (RDP) classifier [36,37], which implements a naive Bayesian classifier method to assign a taxonomic classification at successively broader taxonomic levels if the read is similar to more than one reference sequence (using *assignTaxonomy* function with default values, except for minboot which was set at a more conservative value of 70 instead of the default of 50). Reference libraries were created using the SILVA database (version 138 SSU Nr99) [38]. Reference library creation included filtering based on *in silico* amplification using the *virtualPCR* function from the insect package version 1.4.2 [39] and marine taxonomy was corrected based on world register of marine species [40] using WORRMS package version 0.4.2 [41]. The code used to create reference libraries (https://zenodo.org/badge/latestdoi/134130931), DADA2 pipeline (https://zenodo.org/badge/latestdoi/242739654) and to run analyses and create plots are publicly available (https://zenodo.org/badge/latestdoi/176895539). Reference databases are archived here: for 18S_V7- https://figshare.com/articles/dataset/SILVA_138_trimmed_euka02_dada2_names_fasta/23694339; and 18S_V9- https://figshare.com/articles/dataset/SILVA_138_trimmed_18smini_dada2_names_fasta/23694345. Metadata for samples is available here: https://figshare.com/articles/dataset/Arctic_surface_data_all_pub_xlsx/23694300.

We took additional steps to reduce false positives from sequencing and minimize sequences associated with contamination. First, we removed any ASV that only occurred in one sample. Second, the greatest number of reads within any of the blanks was determined for each ASV and the sum of the reads from all these ASVs was calculated – blank sum. ASVs were removed that had more than 0.001 of reads in any blank compared with the blank sum. For example, if the blank sum was 230 000 reads, then any ASV with more than 230 reads in any of the blanks was removed from all samples. Third, ASVs were removed if the number of reads were less than 0.0001 of the total reads of all samples within a library. For example, any ASV with less than 100 total reads was removed if there were 10 million reads in all samples. The purpose of these filter parameters was to remove false positives caused by contamination and sequencing errors, while allowing for a small amount of cross-contamination (e.g. from tag jumps) to reduce false negatives. Fourth, an ASV was removed if it was not assigned to either a family genus or species, or if it was not assigned to either a phylum, class or order. Fifth, all ASVs were assigned clusters using the cluster_otus function (similarity = 0.97) in the seqateurs package version 0.0.0.9000 [42] and the reads of ASVs with identical taxonomy and cluster were summed for each primer pair. These last two steps aimed to remove spurious ASVs and make results more comparable to species. Given we did cluster ASVs we will refer to them as Operational Taxonomic Units (OTUs) from now on. Finally, to minimize the effect of low read counts per sample on our findings we removed samples that had less than 50% of the lower quartile of the reads of samples for each primer pair. To minimize the effect of different sequencing depths among samples, the reads were rarefied for each sample (lower quartile of all samples used as rarefying number) and the per cent for each OTU was calculated for each sample. Unless otherwise noted, data from the primer pairs was combined by taking the mean per cent of rarefied reads. Only samples with both primers after passing all filter steps were included. This maximized the benefits of using two primer pairs by reducing potential biases of an individual primer pair, including biases associated with PCR amplification and biases resulting from taxa being present or absent in the reference library.

### (e) Environmental variables

Environmental variables were chosen *a priori* and included water depth, bottom temperature, sea surface temperature (SST) change, distance to land, primary productivity, photosynthetic active radiation (PAR), nitrate and ice cover. The mean bottom temperature, ice cover (fraction of year that a location is covered by ice), nitrate, PAR and primary productivity were extracted from Bio-ORACLE layers which are based on satellite data from 2000 to 2014 [43]. Distance to land was obtained from the global marine environment datasets [44]. The SST change was the decadal linear change in SST (Had1SST) calculated from the mean annual SST from 1980 to 2016 using the Met Office Hadley SST data [45] calculated with the load_hadsst function from the *hadsstr* package [46]. Data from each global spatial layer was extracted for each sample location using the extract function from the *raster* package in R [47]. If data did not exist for the point in any layer, the data from the closest cell was extracted for that location.

We then removed variables that had high multicollinearity (variance inflation factor greater than 4) [48] with preference to variables previously found to relate with Arctic marine fauna. Specifically, ice cover was removed because it was related to bottom temperature, nitrate was removed because it was related to primary productivity and distance to land (maximum distance was 162 km) was removed because it was related to surface temperature change (electronic supplementary material, figures S1, S2). It should be kept in mind that we are assessing associations, and drivers cannot be disentangled when variables are related (e.g. bottom temperature and ice cover).

### (f) Statistical analysis

We assessed the relationship between environmental variables and the eDNA community using multivariate generalized linear modelling (MGLM; [49]). GLM is similar to distance-based multivariate analyses, such as PERMANOVA, but is more statistically explicit because the fit of the model to the data can be assessed through evaluating relationships among data, residuals and fitted values, and the mean-variance relationships can be specified depending on data distribution [49,50]. Predictor variables were centred and scaled (mean subtracted and divided by standard deviation) to reduce multicollinearity and reduce difference in magnitude and variance among variables [48]. The presence/absence of each taxa for each sample was the dependent variable because of the biases associated with metabarcoding and unknowns when combining multiple primer pairs. To remove the influence of eDNA from terrestrial species, non-marine species were removed from the dataset (species not categorized by the WORRMS database as marine or estuarine; seven species removed). We used the *manyglm* function within the mvabund package version 4.2.1 with a binomial distribution with cloglog link. The model fit was appropriate with a linear quantile plot and no clear pattern between residuals and sample or taxa was found. Variable significance was determined using the Wald statistic with 1000 permutations using the *ANOVA* function in the mvabund package with the score test and cor.type = ‘shrink’ to account for some correlation among samples. A step-down resampling procedure accounted for multiple tests when calculating the significant association between environmental variables and individual taxa. The samples were not evenly dispersed through space, which could affect their independence if there is spatial autocorrelation. However, comparing the community similarity with the distance between samples (pairwise comparison of all samples) did not indicate that closer samples had more similar communities than samples taken farther apart (electronic supplementary material figure S3). Three additional multivariate GLM analyses were run to assess potential biases of our dataset. This includes separate models for the two different eDNA preservation methods (RNAlater and DNAgard) and when terrestrial species were included in the dataset.

We did not include latitude in the MGLM analysis because latitudinal patterns are probably driven by related environmental variables (latitude was correlated with temperature) [51]. However, to assess how latitude related to changes in the eDNA community and assess if and how this association deviated from a linear relationship, we visualized the relationship and linearity between five environmental variables and the community using Nonmetric Multidimensional Scaling (NMDS) using the Bray–Curtis similarity matrix on presence/absence data. NMDS was calculated with the *metaMDS* function and the *ordisurf* function to plot the general additive model based on thinplate splines with cross-validatory selection of smoothness within the vegan package version 2.6.4 [52]. Finally, we included an NMDS to look at the patterns associated with the sample preservation method, collection method and sample campaign.

## 3. Results

### (a) Sequencing overview

After demultiplexing the samples and removing primers, the two primer pairs, sequenced in separate MiSeq runs, had a similar number of reads per sample with 18S-V7 having 150 218 ± 8467 (mean ± standard error) and 18S-V9 having 138 100 ± 3237. After the DADA2 pipeline, the read count per sample was reduced to 126 412 ± 7216 and 85 075 ± 2573 for the V7 and V9 regions, respectively (electronic supplementary material, table S3). After removing OTUs that were only in a single sample, OTUs common in blanks and with a clear taxonomy assignment (majority of taxonomic levels assigned), the V7 primer pair had 27 933 ± 3560 reads per sample and the number of unique OTUs declined from 165 ± 7 to 51 ± 2 per sample (electronic supplementary material, table S4). The step removing the most reads and unique OTUs was removing OTUs without a clear taxonomy. The effect of removing OTUs based on filtering steps including the number of reads, occurrence in more than one sample and occurrence in blanks was different for the V9 primer pair; with many reads being removed when accounting for OTUs in blanks and OTUs with clear taxonomy; the number of reads per sample after filtering was 14 753 ± 1485 and the number of unique OTUs per sample was 57 ± 21. Read counts in the PCR blanks were low compared with the extraction blanks, which was exemplified by the V7 primer pair which had a mean of 68 665 reads for the two extraction controls but only 1431 reads in the PCR control. We made many attempts to reduce amplification in extraction controls including reordering all reagents, and the reagents used in this extraction protocol can lose effectiveness if decontaminated in a muffle furnace. However, taxa that were removed during the blank filter step did not give a clear indication of the source and differed between the primer pairs. For example, the contaminants for the V7 primer pair were primarily Fungi, making up both 50% of the reads and ASVs removed, while contaminants for the V9 primer pair were primarily Chromista which made up 75% of the contaminant ASVs and unknown Chromista ASVs made up more than 25% on the contaminant reads. Finally, positive amplification does occur in extraction controls in other studies using eDNA (Sigsgaard *et al*., 2017 [53]). But after filtering based on multiple controls and multiple other filtering steps, the number of reads in the controls was a small fraction of the reads in the samples (less than 0.3 and 8% for the V7 and V9 regions, respectively), which could be expected based on tag-jumps during sequencing.

### (b) Overall patterns

Overall, two phyla dominated the abundance of reads, Ochrophyta (including diatoms and brown algae, among others) and Myzozoa (including many protozoans), when primer pairs were pooled (figure 1). These two phyla were followed by Cercozoa (a group of single-celled eukaryotes), Arthropoda (invertebrates including crustaceans) and Ciliopora (protozoans with cilia), which were the 3rd through 5th most abundant phyla. Other groups that were prevalent in the samples included algae (chlorophyta, rhodophyta, haptophyta), worms (annelida), chordates and echinoderms.

**Figure 2.**
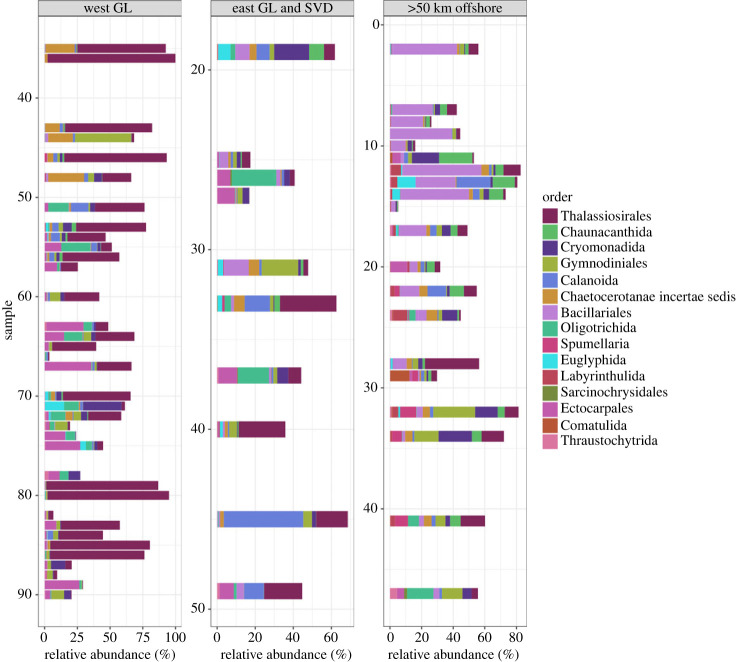
The relative abundance of the most prevalent orders for locations on the west coast of Greenland and locations on the east coast of Greenland and near Svalbard. Samples are numbered from north to south and are shown in figure 1*a*. Blank spaces along the *y*-axis were kept to maintain north to south sample labels.

Whether collected on the west coast of Greenland, the east coast of Greenland and Svalbard, or collected from greater than 50 km from shore, the samples were dominated by similar phyla. (Figure 1). However, the west coast of Greenland had greater abundances of Ochrophyta compared with the other locations. Results for individual orders indicated that Thalassiosirales, an order of diatoms within Ochrophyta, was the most abundant order, especially along the west coast of Greenland (figure 2). Chaunacanthida, in the phyla Radiozoa (single-celled radiolarians), was the second most abundant order and was most prevalent in the northern offshore samples. The northern offshore samples also had a greater abundance of the order Bacillariales, another order of diatoms in Ochrophyta, while there was a relatively greater abundance of Ectocarpales, an order of brown macroalgae in Ochrophyta, in the central area along the west coast of Greenland (figure 2).

There were also patterns in the animal taxa identified, and these groups are often the focus of traditional studies (electronic supplementary material, figure S4). Calanoida (copepod) and Phyllodocida (polychaete) were more prevalent in northern samples compared with southern locations. The opposite pattern was indicated for Monhysterida (nematode) and Podocopida (ostracod), which were more prevalent in the south compared with the north. There were no animal orders that were clearly more prevalent offshore, although a few offshore samples were dominated by terrebellids (a genus of polychaete worms, ‘spaghetti worms’).

### (c) Patterns among primer pairs

At a broad taxonomic level, the primer pairs had a similar number of unique phyla with 18S-V7 having 22 and 18S-V9 having 20. However, there were phyla detected by one primer and not the other. Seven phyla were detected by the V7 and not the V9 primer pair and included Ciliophora, Echinodermata, Cryptophyta, Nemertea, Choanozoa, Ctenophora and Ascomycota. Conversely, the V9 pair identified five phyla that the V7 did not, which included Basidiomycota, Rhodophyta, Tracheophyta, Bryozoa and Platyhelminthes. There was some agreement among the phyla with the most reads and the two primer pairs had four of the same phyla in the top five most abundant phyla for each primer pair (electronic supplementary material, figure S5). Within the 15 most abundant phyla for each primer pair, 11 were common to both primer pairs. Differences between the primer pairs were more pronounced at lower taxonomic levels. The primer pairs detected 77 and 52 orders for the V7 and V9 regions, respectively. Twenty-nine orders occurred in the data of both primer pairs while the V7 primer pair had 47 orders not present in the other primer pair and the V9 primer pair had 23 orders not present in the other primer pair. When focusing on the orders with the most reads, only two of the orders with the top five most abundant reads for each primer pair were common to both primers, and only six of the orders within the top 15 most abundant reads for each primer pair were common to both primers (electronic supplementary material, figure S6).

### (d) Community associations with environmental variables

**Figure 3.**
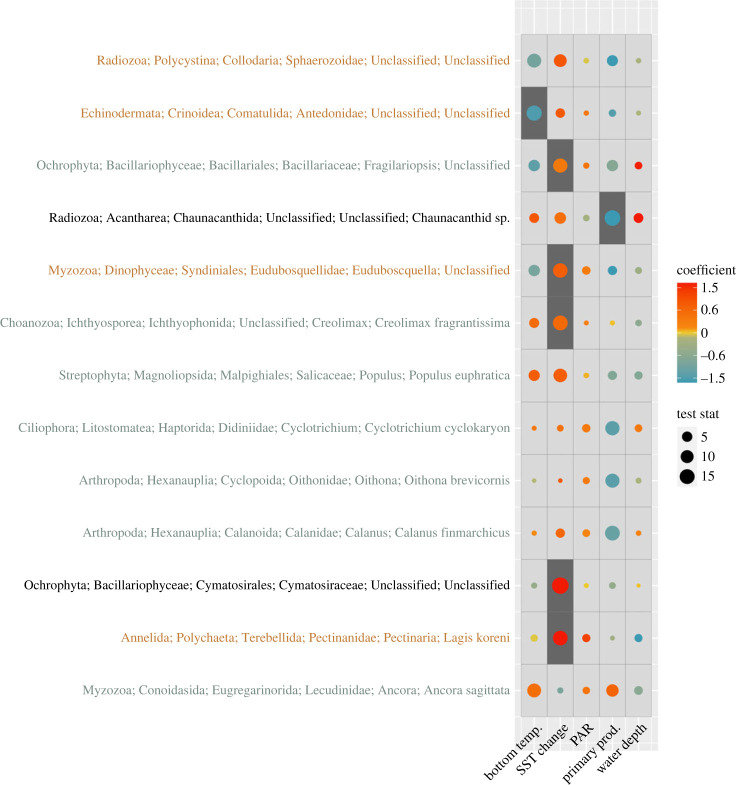
Results of the multivariate general linear models for eukaryote assemblages assessed by eDNA. Taxa order and colour along the *y*-axis indicate clusters based on the coefficients between the taxa and the independent variables. Significant variables (*p* < 0.05) are indicated by a dark background and were adjusted for multiple tests. Magnitude of the test statistic is shown by the size of the circles and the relationship between species and variables (coefficient) are shown by the colour of the circle. Taxa shown had *p* < 0.10 in at least one of the five variables. Taxonomy is shown to genus because of potential ambiguity in species assignment for these broad 18S primers.

Temperature explained the most variation in the eukaryotic communities when all variables were included in a multivariate GLM followed by SST change, PAR, primary production and depth (table 1; based on Wald value), and temperature, SST change and primary production were significant. Across the study area, a feather star in the family Antedonidae had a negative relationship with bottom temperature (figure 3). Five different taxa had a positive relationship with change in SST over the past four decades (increasing with warming), including two taxa within Ochrophyta (diatoms), one within Myzozoa (dinoflagellate), one within Choanozoa (parasitic eukaryote) and a Terebellid worm. A radiolarian and a calanoid copepod showed a negative relationship with primary productivity while water depth and PAR had no significant relationship with any specific taxa. Additional multivariate GLM models indicated no clear bias in the associations between the community and the environmental variables for when the model included samples preserved in DNAgard compared with RNAlater (electronic supplementary material, table S4). In addition, the model with non-marine species removed was almost identical to the overall model (table 1 compared with electronic supplementary material, table S5).

**Table 1.** Summary of the multivariate general linear model assessing the association between the eDNA eukaryote community and environmental variables.

variable	Res. DF	d.f.	Wald value	*p*-value
bottom temperature	64	1	89.78	<0.001
SST change	63	1	101.1	0.003
PAR	62	1	108.07	0.099
primary productivity	61	1	123.8	0.03
water depth	60	1	127.46	0.358

**Figure 4.**
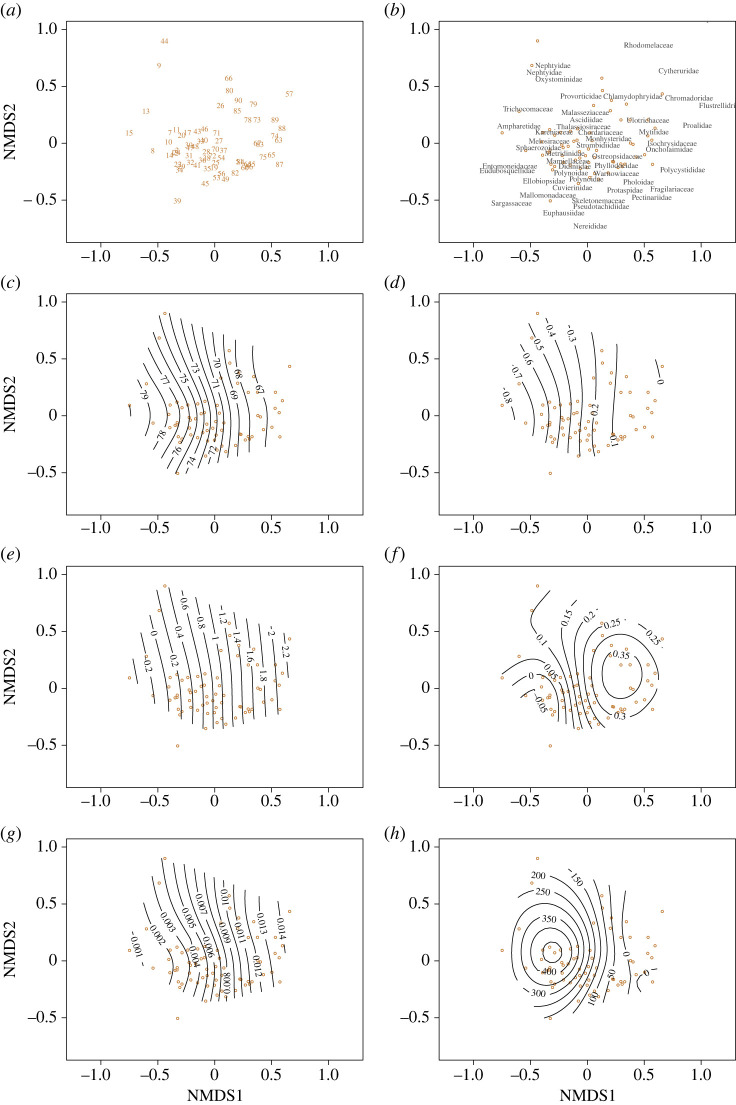
The similarity of sampled eDNA communities (*a*), with sample number corresponding to locations in figure 1*a*, calculated using the Bray–Curtis similarity matrix on log transformed per cent abundance and displayed using NMDS. Identified taxa, labelled with their families, overlaying the samples (*b*) and the smoothed surfaces calculated by GAMs of latitude (*c*), (degrees north), sea ice cover (*d*), (% of year), bottom temperature (*e*), (°C), SST change (*f*), (°C*decade^−1^), primary productivity (*g*), (g*m^−3*^day^−1^) and water depth (*h*,*m*). Stress, a measure of fit, was 0.091 for the NMDS. Parallel surfaces indicate linear relationships, while curved and unevenly spaced surfaces indicate nonlinear relationships between variable and eDNA community.

To assess if the relationships between eukaryotic community and environmental variables were linear we also ran individual Generalized Additive Models (GAMs) for each variable. Sea ice cover explained the most variation in communities (71%) followed by latitude (69%), SST change (58%), primary productivity (49%), depth (42%) and bottom temperature (34%; table 2). The high amount of variation explained by each individual variable indicates that some of the variables are related themselves and overlap in their ability to explain the variability in the eukaryote community which is why they were not all included in the single, multivariate GLM. Latitude, sea ice cover, bottom temperature and primary production all exhibited patterns similar to a linear relationship with eukaryote communities as suggested by parallel contour lines (figure 4). SST change indicated a nonlinear relationship, particularly around samples collected off the east coast of Greenland. Depth had a clear nonlinear relationship with eukaryote communities which was primarily influenced by samples off the northeast coast of Greenland. Stress for all plots were less than 0.09 indicating that the reduced dimensions of the plots were a good representation for the data. Finally, non-dimensional scaling did not indicate any clear differences in the variation of communities for samples collected using a corer or scoop/grab, particularly when considering the differences in the number of samples, sampling campaign and preservation method (electronic supplementary material, figure S6).

**Table 2.** Summary output of individually run General Additive Models. Independent variable is listed and the dependent variable was the eDNA community.

variable	Est. DF	Ref. Df	*F* value	*p*-value	deviance explained (%)
latitude	5.86	9	16.73	<0.0001	71.6
sea ice cover	5.58	9	14.21	<0.0001	69.2
bottom temperature	2.59	9	3.24	<0.0001	33.7
SST change	5.50	9	8.61	<0.0001	58.2
primary productivity	4.42	9	6.17	<0.0001	49.7
water depth	4.76	9	4.46	<0.0001	41.5

## 4. Discussion

We conducted a large-scale, eDNA-based survey using sediment samples collected around Greenland and Svalbard to assess the diversity of Arctic marine communities. Two different eukaryotic primer pairs detected 27 phyla and 99 orders. The importance of running multiple 18S markers when assessing diversity became more evident at lower taxonomic levels (order) as the overlap between the markers becomes minimal, so the use of two primer pairs improves detection. Using eDNA along with other traditional methods could improve our understanding of Arctic diversity both across space and time given the difficulties in sampling these coastal and oceanic ecosystems [54].

Changes in the communities through space indicated that bottom temperature, SST change, primary production, latitude and sea ice cover were the variables most strongly related to changes in eukaryotic communities. These results highlight the underlying potential effect of warming and ice loss in altering Arctic communities, which is in agreement with organism-based surveys [55,56].

### (a) Methodological considerations of eDNA-based surveys

Assessing communities using eDNA can assist in creating baseline data and in understanding current and future impacts of climate change on biodiversity patterns in the Arctic. However, there are considerations when interpreting results from eDNA-based studies. A primary consideration is minimizing and accounting for potential contamination and false positives. A primary way to do this is by including controls. We did include extraction and PCR blanks and then removed taxa based on read abundances within these controls. It is best practice to also include field controls to account for any contamination when collecting samples. For water samples this is relatively straightforward and includes processing DNA-free water just as if it were a true sample. However, it is more complicated to include field control for sediment samples and a recent review found no sediment eDNA studies used field blanks [57]. We do recommend studies on sediment eDNA including field blanks, perhaps including sterilized sand but further improvements need to be considered for this issue. Given these difficulties, we were not able to include field blanks. One way to assess contamination in the field and in general, is to compare documented distribution of species with eDNA-based detections. A separate study using the same data as this study found that detections of macroalgal orders from eDNA aligned well with documented distributions, and detections of seagrass only occurred at locations near known meadows [58]. Although this is only a subset of eukaryotes detected, it does indicate our results align with known distributions.

The ability to assess communities from diverse habitats is a benefit of using eDNA, for which sediment type does not likely affect DNA extraction [30]. However, our sediment collection methods did differ among sites given that the same method could not be used across all habitats. For example, intertidal methods could not be used in deep water. There are potential biases associated with using different collection methods particularly with regards to disturbance of sediment and preservation method. Although we cannot rule these out, it is unlikely that this affected our conclusions given that we chose sampling methods and protocols that specified that eDNA samples needed to be from undisturbed collected sediment. It should also be noted that the community detected in eDNA within sediment could represent the community over longer time periods than water and this is dependent on many variables including preservation, depth of the sample and the accretion rate [59,60]. However, eDNA in sediment does have exponential degradation rates (similar to eDNA in water), indicating that detections are more likely recently present species [59].

Diversity assessments using eDNA have compared multiple primer pairs. For example, a study on coral reefs showed that using multiple primers successfully detected taxa across the tree of life on a coral reef and an 18S V4 primer pair detected the most taxa, more than an 18S V1–V3 primer pair [61]. In addition, an 18S V4 primer pair was found to be better than two other 18S primer pairs for detecting change in sediment eDNA caused by oil platforms in the Adriatic Sea [62]. We found that using multiple primers becomes ever more important as comparisons between them include more specific taxonomy. A majority of the 27 phyla were detected by both primer pairs and 12 were unique to one of the primer pairs, but of the 99 orders detected, 70 were unique to only one primer pair. These differences were also apparent when considering read abundance as only six of the most abundant 15 orders were detected by both primer pairs. Our results do assist in primer choice depending on a study's target groups. For example, the V7 primer pair detected more microorganisms such as ciliates, choanoflagellates and cryptophytes, while the V9 primer pair should be used to detect red algae and bryozoans. If a study is restricted by resources and can only use one primer pair to assess biodiversity, then the V7 primer pair detected a broader array of taxa than the V9 primer pair (detected 77 compared with 52 orders, respectively).

Studies are usually restricted by resources which limits the usage of multiple primer pairs but also the number of replicates. Sequencing replicate samples can increase the number of species detected [20,63], especially if taxa have low read abundance, and can be used to filter results to reduce the potential of false positives [64]. The primary goal of this study was to assess overall diversity and its drivers, and we prioritized using two primer pairs to detect the broadest array of taxa and did not include multiple replicates. It is important for researchers to consider their priorities and study goals when deciding the most appropriate study design and whether to include replicates and/or multiple primer pairs.

### (b) Environmental drivers of Arctic biodiversity and space-for-time predictions

Our assessment of a broad array of eukaryotic taxa and the relationship of these communities with environmental variables found that temperature is a primary driver of changes in Arctic marine communities with bottom temperature and SST change explaining the most variation in an overall model and sea ice cover, latitude and SST change each explained more than 50% of the variation in the community when run individually in nonlinear models.

Global warming is predicted to increase surface and bottom water temperature and reduce sea ice cover [65], and likely have significant impacts on Arctic communities. Our study location across space contained a wide variation in the environmental variables spanning 16° latitude (64–79), 5.3°C bottom temperature (−1.5 to 3.8°C), 0–0.3°C change in SST per decade and 0–70% annual ice cover. These same four variables had very similar associations with Arctic communities as indicated by the nonlinear multivariate models (figure 5), suggesting that changes in these communities are driven in a similar direction by all these temperature-related variables. Utilizing these data along with our eDNA-based survey across space enabled inferences about temporal changes in Arctic communities under climate change. Multiple IPCC warming projections for the Arctic are greater than the temperature range across our sites, which suggest greater differences could occur in Arctic communities over the coming decades than we measured over 14° latitude, which will likely result in profound changes in the overall community including addition of warm-tolerant species and loss of cold-tolerant species.

### (c) Indicator taxa

Given the effect of climate change and its associated consequences on Arctic communities, it is important to identify and observe taxa that are indicative for a habitat and the environmental conditions therein, since they can act as indicators for broader ecosystem changes. Highly specialized or environmentally restricted organisms may struggle to adapt to changes in their preferred ecosystem or even loss of habitat [66,67]. The highly variable sea ice habitat, for example, is hard to access and sampling efforts there are time consuming, which results in under-sampling and only scarce coverage and identification of habitat-specific species [68].

We found multiple taxa groups that were significantly associated with specific environmental variables (figure 3). However, only a few were identified to an appropriate lower taxonomic level so the corroborative data could be found. Five different taxa had a positive relationship with change in SST over the past four decades (increasing with warming), including two diatoms (Kingdom Ochrophyta), one dinoflagellate (Kingdom Myzozoa), one parasitic eukaryote (Kingdom Choanozoa) and a bristleworm (Kingdom Annelida). The diatoms were classified to genus and family, which include species from many regions and thus the potential as indicator taxa remains unknown. *Creolimax fragrantissima* is a eukaryote parasite of invertebrates documented in the Pacific and both its range and the prevalence of similar species are relatively unknown [69]. Thus, it is possible that this species or a close relative is an indicator species, but more research is needed. Another potential indicator species was the Terrebellid worm, *Lagis koreni*. This bristleworm is common in northern Europe [70] and seems like a good candidate for an indicator of global warming. In general, research to support or contradict our findings was scarce, which highlights the limited knowledge of these communities. It is also important to note that very broad primers and incomplete reference libraries were used, and caution should be taken when focusing on specific species detected. For example, we detected a poplar tree (*Populus euphratica*; not included in analyses) which inhabits temperate areas, however, there are species with this Populus genus that inhabit Greenland. This indicates a misassigned species probably because of the incomplete reference library. More studies on relationships between Arctic communities and environmental variables, as well as better reference libraries of Arctic eukaryotes will improve the ability of eDNA to be used to identify indicator species and sequences should be archived for future studies to re-analyse.

## 5. Conclusion

We detected eukaryote DNA from a total of 27 phyla and 99 orders in Arctic marine surface sediments, reflecting current presence of these taxa across gradients of environmental conditions. The use of complementary primer pairs was a clear advantage as the combination of the two studied 18S primers (V7 and V9) resulted in a more complete inventory of species than obtained by a single primer pair. The identified current distribution patterns of marine biodiversity can serve as a baseline for future studies to monitor potential changes. The distribution patterns also informed predictions of biodiversity responses to climate change via space-for-time approaches and via identification of indicator taxa. Hence, spatial analyses linked the presence of a number of taxa to water temperature, changes in water temperature and/or the extent of sea ice cover, suggesting that future warming and loss of sea ice might generate parallel changes. Overall, our findings highlight that eDNA analyses of marine sediments can be a tool to supplement the assessment of marine biodiversity, as well as to diagnose possible environmental changes, in remote Arctic regions where observational records are scarce and difficult to obtain.

## Data Availability

All raw sequences are available on National Center for Biotechnology Information (NCBI) under Bioproject PRJNA996092. Code used for analyses is available at https://github.com/ngeraldi/eDNA_Arctic_surface-sediment and archived to create reference libraries (http://dx.doi.org/10.5281/zenodo.8153426), DADA2 pipeline (http://dx.doi.org/10.5281/zenodo.8153415) and to run analyses and create plots are publicly available (http://dx.doi.org/10.5281/zenodo.8153430). Reference databases are archived here: for 18S_V7- https://figshare.com/articles/dataset/SILVA_138_trimmed_euka02_dada2_names_fasta/23694339; and 18S_V9- https://figshare.com/articles/dataset/SILVA_138_trimmed_18smini_dada2_names_fasta/23694345 . Metadata for samples is available here: https://figshare.com/articles/dataset/Arctic_surface_data_all_pub_xlsx/23694300. Supplementary material is available online [71].
